# The *Wx*/*SSIIa* and *GS3*/*GW7* Alleles, Both Individually and in Combination, Can Significantly Distinguish Rice Germplasm Quality

**DOI:** 10.3390/ijms26146726

**Published:** 2025-07-14

**Authors:** Yuanyuan Hao, Junfeng Xu, Mingming Wu, Ying Zhu, Jiayu Song, Yifei Han, Chunshou Li, Fudeng Huang

**Affiliations:** 1 Institute of Crop and Nuclear Technology Utilization, Zhejiang Academy of Agricultural Sciences, Hangzhou 310021, China; haoyuanyuan@zaas.ac.cn (Y.H.); wumm@zaas.ac.cn (M.W.); 15940148227@163.com (J.S.); hanyf@zaas.ac.cn (Y.H.); lichunshou@126.com (C.L.); 2 Key Laboratory of Traceability for Agricultural Genetically Modified Organisms, Ministry of Agriculture and Rural Affairs, Zhejiang Academy of Agricultural Sciences, Hangzhou 310021, China; njjfxu@163.com; 3 State Key Laboratory for Quality and Safety of Agro-Products, Zhejiang Academy of Agricultural Sciences, Hangzhou 310021, China; yzhzaas@163.com

**Keywords:** rice quality, *Wx*, *SSIIa*, *GS3*, *GW7*, allelic combination

## Abstract

As living standards rise, there has been a growing emphasis on quality traits related to rice’s taste potential. Recent studies explored correlations among quality traits, but the influence of major genes governing a specific quality trait on other quality traits remains elusive. Here, we report on the application of grain quality genes, two of *Waxy* (*Wx*) and *Starch synthase II-a (SSIIa)*, which dominates in rice cooking and eating quality, and two grain length/width-related genes, *grain size 3* (*GS3*) and *grain width 7* (*GW7*), on appearance quality traits. Five allele-specific markers for these genes were developed, and used to stack the desirable alleles at these loci. The effects of individual or combined alleles at the loci were evaluated using a set of 156 rice germplasm. We found that the Wx-In1 (Intron 1) locus exerts a major effect in controlling both amylose content and gel consistency, while the SSIIa-Ex8 (Exon 8) locus primarily governs alkali spreading value. The impact on chalkiness-related traits follows the hierarchy of Wx-In1 > SSIIa-Ex8 > Wx-Ex10 (Exon 10). GS3-Ex2 (Exon 2) has a highly significant impact on chalkiness-related traits, and the GW7-Pro (Promoter) locus exerts a synergistic effect. The GS3-Ex2 locus exerts an effect in controlling both gel consistency and alkali spreading value, while the GW7-Pro locus governs amylose content. The data for newly developed allele-specific markers will facilitate the improvement of rice quality in rice.

## 1. Introduction

Rice serves as the primary food source for approximately one-third of the global population, with China standing as the preeminent producer and consumer. Consequently, achieving high yields and good quality in rice cultivation has always been paramount [1]. As living standards rise, there has been a growing emphasis on quality traits related to rice quality, including milling quality, appearance quality, cooking and eating quality (CEQ), and nutritional quality [2]. Notably, appearance quality and CEQ occupy a particularly significant position among these traits.

Starch, the primary constituent of rice endosperm, is primarily composed of amylose and amylopectin, two distinct forms that contribute to the unique physicochemical properties of the grain [3]. The physicochemical properties of starch, including the amylose content (AC), gel consistency (GC), gelatinization temperature (GT), have been utilized to evaluate rice CEQ [4], where AC is negatively related to taste palatability, including transparency, viscosity, and rice milling quality, considered to be the predominant attribute of CEQ [4]. GT is positively correlated with physical characteristics responsible for cooking time and the ability to absorb water during cooking [5]. Rice grains with high GT require more water and cooking time than rice with low or medium GT. GT can be measured indirectly in terms of an alkali spreading value (ASV) using an alkaline or urea solution. GC is analyzed by measuring the cold paste viscosity of cooked milled rice flour [6]. Rice with a soft GC is highly preferred among consumers [6].

The major genes responsible for controlling AC, GC, and GT have been cloned. The *Waxy* (*Wx*) gene encoding granule-bound starch synthase I (GBSSI) is the major determinant of AC and GC, and a minor contributor to GT [7]. *Wx* considered to be the first target of selection during both the domestication and subsequent crop diversification of rice. Numerous allelic variations in *Wx* (*Wx^lv^*, *Wx^a^*, *Wx^in^*, *Wx^b^*, *Wx^mw^*, *Wx^mp^*, *Wx^mq^*, *Wx^op/hp^*, and *wx*) have contributed to the regional variation in rice AC and have affected consumer preferences [8]. *Wx^a^* predominates in indica cultivars with high AC. *Wx^a^* produces OsGBSSI protein levels approximately 10 times higher than *Wx^b^*, which is primarily distributed in japonica cultivars exhibiting low to intermediate AC [9]. This disparity in OsGBSSI abundance stems from a G-to-T mutation at the splicing donor site of *Wx^b^* intron 1 [10]. Additionally, a C-T substitution on exon 10 of *Wx* is considered the major control of GC [11]. However, association analysis showed the *Wx* gene only accounts for nearly 40% of phenotypic variation for GC [3], some varieties with the same level of AC exhibited different GC [12], and until recently, only one quantitative trait locus (QTL), *qGC10* (not a *Wx* allele), had been shown to contribute to GC [13]. *Starch synthase II-3* (*SSII-3*, also known as *ALK* and *SSIIa*) is a major gene regulating GT, and it also contributes to AC and GC [14]. The GC/TT substitution on exon 8 of *ALK* could differentiate rice with high or intermediate GT from those with low GT in about 90% of cases [15,16]. *Wx* and *ALK* genes are closely located on chromosome 6; hence, they have been co-selected during rice domestication [3].

Grain appearance is mainly determined by grain shape and opacity. Opacity includes chalkiness and transparency, while grain shape is described by grain length, width, and thickness, which refers to both grain yield and quality [17]. Both grain shape and grain opacity are quantitative traits controlled by QTL, and are largely influenced by the environments. Dozens of genes conferring grain shape and/or grain chalkiness were cloned in rice, using map-based cloning strategies [17]. For example, a grain size gene, *grain size 3* (*GS3*), is the most important regulator of grain length in both natural and breeding populations of cultivated rice. Its natural variants, the C to A nucleotide substitution in the second exon of *GS3*, leading to long-grain length, have contributed greatly to the improved productivity and grain quality of rice on a global scale [18,19]. The grain-associated gene, *grain length 7/grain width 7* (*GL7/GW7*), which encodes a TONNEAU1-interacting motif protein, plays a pivotal role in imparting slender grain morphology and mitigating grain chalkiness [20,21]. Notably, the synergistic interaction between the *GW7* and *GS3* alleles concurrently enhances rice yield and grain quality [20].

The various quality traits often influence each other reciprocally. Grain shape demonstrates a significant or highly significant correlation with milling quality [22]. Chalkiness percentage displays a significant negative correlation with rice milling quality and grain length, and a significant positive correlation with grain width and grain weight [23]. Additionally, chalkiness usually affects rice cooking properties with potential low CEQ, as well as the nutritional quality of rice [24,25].

As mentioned above, rice quality traits are closely interrelated, yet the molecular mechanisms underlying their regulation remain largely unknown. In particular, whether major-effect genes controlling one trait may also influence other traits has not been investigated. Given that *Wx* and *SSIIa* are major genes regulating CEQ, while *GS3* and *GW7* are major genes controlling grain shape, with all four genes being expressed in developing endosperm, this study examined the effects of *Wx* and *SSIIa* on appearance quality, and the impacts of *GS3* and *GW7* on both CEQ and appearance quality, aiming to elucidate the molecular basis for the significant correlations among quality traits. A total of 156 rice germplasm samples were used as materials to verify molecular markers for genotyping *Wx*, *SSIIa*, *GS3*, and *GW7*. The impact of allelic variations and allelic combinations of these genes on CEQ and appearance quality indices was analyzed, thereby providing a theoretical basis for the breeding of new rice varieties with superior palatability.

## 2. Results

### 2.1. The CEQ Measured Values of 156 Germplasms Exhibit a Broad Range of Distributions

**Figure 1:**
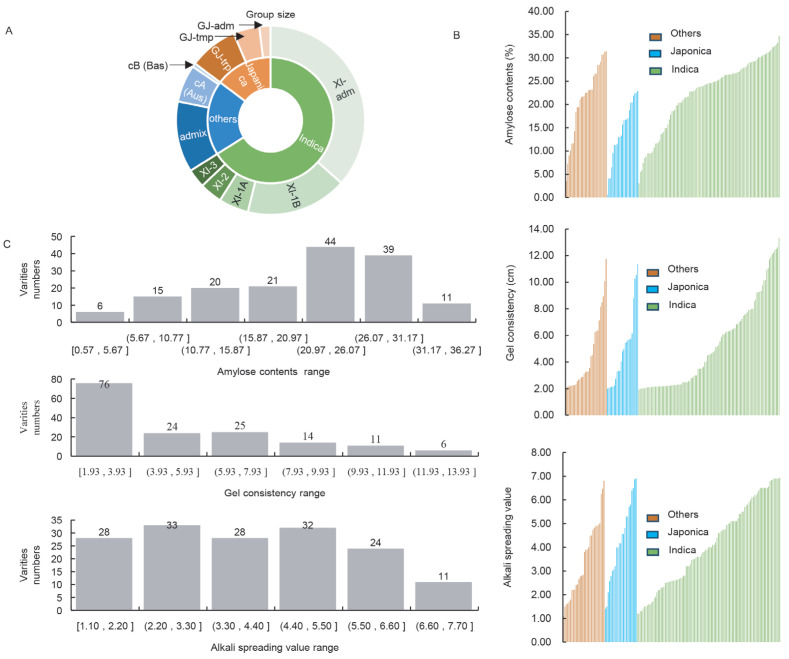
Distribution of CEQ indices among 156 germplasms. (**A**) Distribution of 156 resources across different subgroups. (**B**) Numerical distribution of each CEQ index in indica, japonica, and “others”. (**C**) Distribution of resource counts across different value ranges for each CEQ index.

Based on the classification of germplasm resources from the Rice Functional Genomics and Breeding (https://rfgb.rmbreeding.cn/index, accessed on 25 October 2023), this study selected 156 resources for investigation. Among them, 23 were japonica rice resources, 103 were indica rice resources, and 30 were classified as “others”. The indica rice resources were further grouped into five subgroups: XI-1A, XI-1B, XI-2, XI-3, and XI-Adm, while the japonica rice resources were categorized into three subgroups: GJ-Adm, GJ-Tmp, and GJ-Trp. The remaining resources were divided into three subgroups: Admix, cA (Aus), and cB (Bas) (Figure 1A).

After measurement, data on AC, GC, and ASV for each resource were obtained. Specifically, the AC content of indica, japonica, and “others” ranged from 3.04% to 34.72%, 0.57% to 22.86%, and 3.52% to 31.44%, respectively. The GC length was distributed between 1.93 cm and 13.33 cm for indica, 2 cm and 11.35 cm for japonica, and 2.05 cm and 11.75 cm for “others”. The ASV exhibited a diverse distribution ranging from 1.2 to 6.93 for indica, 1.4 to 6.9 for japonica, and 1.1 to 6.8 for “others” (Figure 1B), with continuous distribution observed for each indicator.

Further analysis of the CEQ measurements of the 156 resources revealed that the majority had amylose content ranging from 20.97% to 26.07%, followed by the 26.07% to 31.17% range. For gel consistency length, 76 resources clustered within the range of 1.93 cm to 3.93 cm. The ASV distribution was more widespread, with over 24 resources distributed in each numerical interval except for 11 resources falling within the 6.6 to 7.7 range (Figure 1C). The results presented above demonstrate that the CEQ values of japonica rice, indica rice, and “others” exhibit a broad range of distributions across various intervals, providing an abundant resource for subsequent haplotype analysis.

### 2.2. Development of Functional Markers of Wx-In1, Wx-Ex10, and SSIIa-Ex8

**Figure 2:**
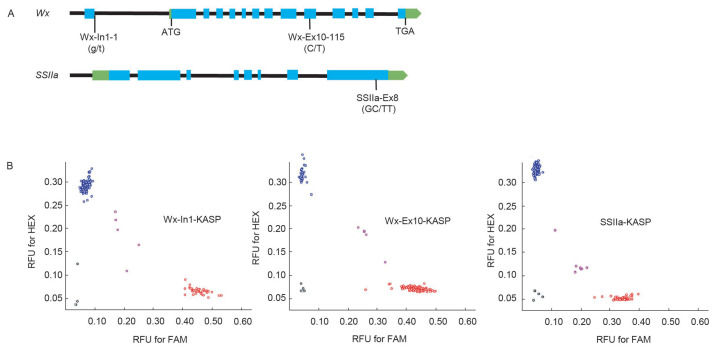
Development of the gene-specific markers Wx-In1, Wx-Ex10, and SSIIa-Ex8 for *Wx* and *SSIIa*. (**A**) Schematic diagram of the respective gene model showing the position of SNPs for the marker development in *Wx* and *SSIIa*. The blue boxes represent exons, and the green boxes represent the 5′ and 3′ untranslated regions (UTRs). (**B**) Genotyping of germplasms using the markers, RFU, relative fluorescence units. The red circles represent the FAM fluorophore, the blue circles represent the HEX fluorophore.

Based on the G/T SNP differences in the first intron (In1) of *Wx*, the C/T SNP differences in exon 10 (Ex10), and the two consecutive GC/TT SNP differences in exon 8 (Ex8) of *SSIIa*, three efficient KASP markers were designed (Figure 2A). These markers accurately categorized the corresponding alleles in 156 germplasm into three distinct groups based on their allelic variations.

At the Wx-In1 locus, 117 accessions exhibited the GG homozygous genotype, 33 accessions were TT homozygous, and 5 accessions were GT heterozygous. Notably, 75% of the germplasm accessions carried the GG homozygous genotype, with a distribution of 80.6% in indica rice, 39.1% in japonica rice, and 83.3% in other varieties. This pattern reflects the prevalent distribution of *Wx^a^* in indica rice and *Wx^b^* in japonica rice. At the Wx-Ex10 locus, 113 accessions were CC homozygous, 38 were TT homozygous, and 5 were CT heterozygous. The CC homozygous genotype accounted for 72.4% of the total germplasm, with a distribution of 59.2% in indica rice, 95.7% in japonica rice, and 100% in other varieties. This data highlights the high conservation of this locus in japonica and “others” germplasm, while suggesting potential evolutionary divergence in indica rice. Regarding the two adjacent SNP sites in SSIIa-Ex8, 100 accessions were GCGC homozygous, 49 were TTTT homozygous, and 7 were GCTT heterozygous. Among them, 64.1% of the germplasm accessions carried the GCGC homozygous genotype, with a distribution of 62.1% in indica rice, 43.4% in japonica rice, and 86.7% in “others” varieties (Figure 2B, Table S2). This once again demonstrates the diversity and variability of different germplasm accessions at specific gene loci.

### 2.3. Wx-In1 Locus Exerts a Major Effect in Controlling Both AC and GC, While the SSIIa-Ex8 Locus Primarily Governs ASV

**Figure 3:**
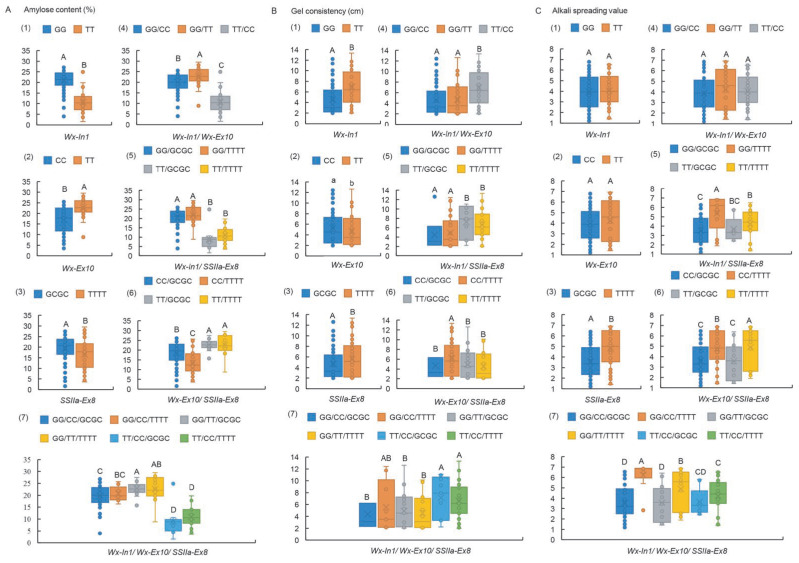
Boxplots showing phenotypic variation of haplotypes assayed by Wx-In1, Wx-Ex10, and SSIIa-Ex8 in rice germplasm. (**A1**–**A7**) AC, (**B1**–**B7**) GC, (**C1**–**C7**) ASV. Box edges represent the upper and lower quantiles, with median value shown as bold line in the box. Internal value points and outlier points are also displayed within the box. A–D indicate significant differences by Tukey’s *t*-tests (*p* < 0.01); a, b indicate significant differences by Tukey’s *t*-tests (*p* < 0.05).

To delve deeper into the individual and combined effects of *Wx* and *SSIIa* genes on the CEQ of 156 rice accessions, a systematic analysis was conducted to quantify the significant variations in CEQ indices across different genotypes and their combinations (Table S4). The results indicated that, for single genes, accessions harboring the Wx-In1-GG, Wx-Ex10-TT, and SSIIa-Ex8-GCGC genotypes exhibited significantly higher AC levels compared to those with Wx-In1-TT, Wx-Ex10-CC, and SSIIa-Ex8-TTTT genotypes, respectively (Figure 3(A1–A3)). These differences reached statistical significance at levels as high as e^−62^, e^−17^, and e^−6^ (Table S3). Within the Wx-In1/Wx-Ex10 genotype combinations, the AC followed the order GG/TT > GG/CC > TT/CC, all at highly significant levels (Figure 3(A4)). Regarding the combinations of Wx-In1/SSIIa-Ex8 and Wx-Ex10/SSIIa-Ex8, variations at the SSIIa-Ex8 locus did not significantly alter the primary effects of Wx-In1 and Wx-Ex10 on AC (Figure 3(A5,A6)), further reinforcing that Wx-In1 and Wx-Ex10 have overwhelmingly significant impacts on AC compared to SSIIa-Ex8. Notably, the influence of SSIIa-Ex8 on AC became significant only when Wx-Ex10 was in the CC genotype (Figure 3(A6)). In trigenic interactions, the Wx-In1 variation remained the absolute primary determinant of AC, followed by Wx-Ex10, while the effect of SSIIa-Ex8 became negligible when interacting with the other two loci (Figure 3(A7)). It is worth noting that accessions with both Wx-In1 and Wx-Ex10 in the TT/TT genotype were not included in this study.

The investigation into the regulatory effects of three key loci on GC revealed that rice accessions harboring the Wx-In1-TT and SSIIa-TTTT genotypes exhibited significantly longer GC compared to those with Wx-In1-GG and SSIIa-Ex8-GCGC genotypes (Figure 3(B1,B3)). Furthermore, accessions with the Wx-Ex10-CC genotype showed an increase in GC length compared to those with the Wx-Ex10-TT genotype (Figure 3(B2)). Notably, the influence of the Wx-In1 locus on GC far surpassed that of Wx-Ex10 and SSIIa-Ex8, with Wx-In1 playing a dominant role in the regulation of GC within the context of two-gene combinations (Figure 3(B4,B5)). For the combination of Wx-Ex10 and SSIIa-Ex8, effective synergistic regulation of GC was only observed when these two loci carried the CC and TTTT genotypes, respectively, which are associated with long GC (Figure 3(B6)). In the context of three-gene interactions, the variation at the Wx-In1 locus had no significant impact on GC only when both Wx-Ex10 and SSIIa-Ex8 loci simultaneously exhibited the CC and TTTT genotypes, which are closely linked to long GC (Figure 3(B7)).

Regarding the impact of the three loci on ASV, the two Wx loci, either individually or combined, do not influence ASV (Figure 3(C1,C2,C4)). However, the SSIIa-Ex8 locus with the TTTT genotype exhibits significantly higher ASV than the GCGC genotype (Figure 3(C3)). Furthermore, only when SSIIa-Ex8 displays the high ASV-associated TTTT genotype do cultivars with Wx-In1-GG and Wx-Ex10-CC genotypes demonstrate significantly higher ASV compared to those with Wx-In1-TT and Wx-Ex10-TT (Figure 3(C5,C6)). This finding aligns with the conclusion of the combined effect of the three genes, and when SSIIa-Ex8 presents the high ASV-associated TTTT genotype, the effects of Wx-In1-GG and Wx-Ex10-CC on ASV exhibit a synergistic effect (Figure 3(C7)).

### 2.4. The Impact on Chalkiness-Related Traits Follows the Hierarchy of Wx-In1 > SSIIa-Ex8 > Wx-Ex10

**Figure 4:**
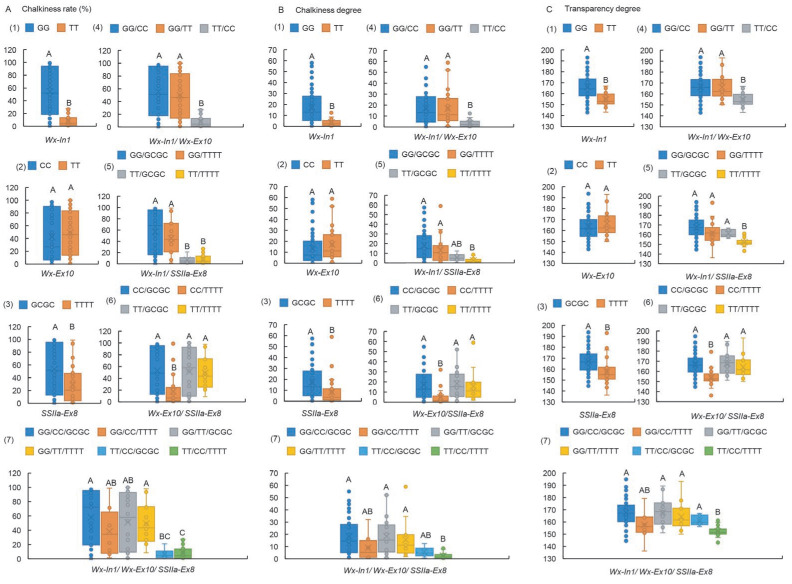
Boxplots showing phenotypic variation of haplotypes assayed by Wx-In1, Wx-Ex10, and SSIIa-Ex8 in rice germplasm. (**A1**–**A7**) CR, (**B1**–**B7**) CD, (**C1**–**C7**) TD. Box edges represent the upper and lower quantiles, with median value shown as bold line in the box. Internal value points and outlier points are also displayed within the box. A–C indicate significant differences by Tukey’s *t*-tests (*p* < 0.01).

The investigation into the effects of three loci of *Wx* and *SSIIa* on rice chalkiness-related traits, encompassing chalkiness degree (CD), chalkiness rate (CR), and transparency degree (TD) (Table S4), revealed that the genotypes carrying Wx-In1-TT and SSIIa-Ex8-TTTT in germplasms CR, CD, and TD showed markedly better performance than those with Wx-In1-GG and SSIIa-Ex8-GCGC (Figure 4(A1,A3,B1,B3,C1,C3)). However, the two alleles of Wx-Ex10 did not significantly influence CR, CD, and TD (Figure 4(A2,B2,C2)). In the combination of Wx-In1 and Wx-Ex10, the difference at the Wx-In1 locus predominantly affected CR, CD, and TD (Figure 4(A4,B4,C4)). Conversely, in the combination of Wx-Ex10 and SSIIa-Ex8, only when Wx-Ex10 was CC did the two alleles of SSIIa-Ex8 have a highly significant impact on appearance quality (Figure 4(A6,B6,C6)). In the Wx-In1 and SSIIa-Ex8 combination, the Wx-In1 locus dominated the influence on CR over SSIIa-Ex8, evident in the combined effects of both loci (Figure 4(A5)), with a similar trend observed in the three-locus combination (Figure 4(A7)). Nevertheless, only when SSIIa-Ex8 was TTTT did the two alleles of Wx-In1 significantly influence CD and TD (Figure 4(B5,C5)). Although the three-locus combination did not reach extreme significance, the influence of Wx-In1 alleles on CD and TD varied based on the SSIIa-Ex8 genotype (Figure 4(B7,C7)). These findings indicate that the impact on appearance quality follows the hierarchy of Wx-In1 > SSIIa-Ex8 > Wx-Ex10.

### 2.5. Development of Functional Markers of GS3-Ex2 and GW7-Pro

**Figure 5:**
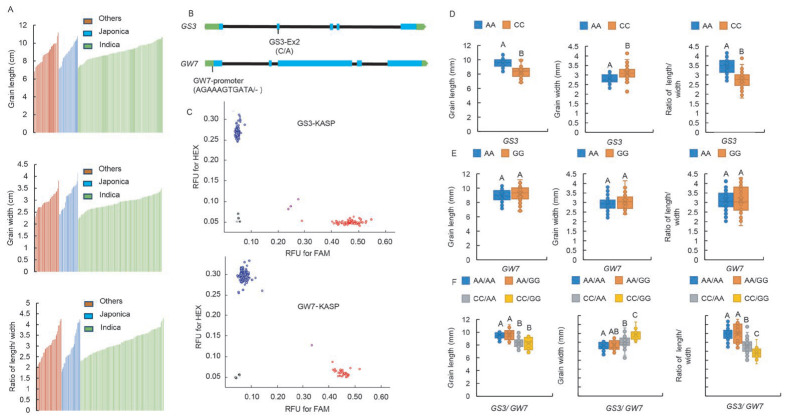
Distribution of grain shape indices among 154 germplasms; design and validation of markers for *GS3* and *GW7*. (**A**) Numerical distribution of each grain shape index in indica, japonica, and “others”. (**B**) Schematic diagram of the respective gene model showing the position of SNPs for the marker development in *GS3* and *GW7*. The blue boxes represent exons, and the green boxes represent the 5′ and 3′ untranslated regions (UTRs). (**C**) Genotyping of germplasms using the markers, RFU, relative fluorescence units. The red circles represent the FAM fluorophore, the blue circles represent the HEX fluorophore. (**D**–**F**) Boxplots showing grain shape variation of haplotypes assayed by GS3-Ex2 and GW7-Pro in rice germplasm. Box edges represent the upper and lower quantiles, with median value shown as bold line in the box. Internal value points and outlier points are also displayed within the box. A–C indicate significant differences by Tukey’s *t*-tests (*p* < 0.01).

Upon measurement, comprehensive data on grain length, grain width, and length-to-width ratio (LWR) were obtained for various resources, including indica rice, japonica rice, and “others”. Specifically, grain length ranged from 6.813 mm to 11.163 mm for indica, 7.128 mm to 10.737 mm for japonica, and 7.177 mm to 10.689 mm for “others”. Grain width exhibited intervals of 2.135 mm to 3.807 mm for indica, 2.421 mm to 4.136 mm for japonica, and 2.207 mm to 3.478 mm for “others”. Similarly, LWR displayed a diverse distribution, spanning 2.03 to 4.26 for indica, 1.8 to 4.24 for japonica, and 2.32 to 4.29 for the rest (Figure 5A). The measured values of all indicators exhibited a continuous distribution pattern (Figure 5A).

Based on the C/A single nucleotide polymorphism (SNP) difference in the second exon (Ex2) of *GS3* and an insertion/deletion (Indel) difference (AGAAAGTGATA/-) in the promoter region (Pro) of *GW7*, two KASP markers were designed and implemented (Figure 5B, Table S1). These markers enabled precise identification of the corresponding alleles in 154 germplasm resources (Figure 5C). These resources were meticulously classified into three groups according to their allelic differences. At the GS3-Ex2 locus, 76 resources exhibited the AA homozygous genotype, 74 were CC homozygous, and 4 were AC heterozygous. Notably, the proportion of germplasm carrying the long-grain AA genotype was 56.4% in indica, 47.8% in japonica, and 26.7% in “others”, indicating a relatively even distribution across resource types. At the GW7-Pro locus, 119 resources were AA homozygous, 34 were GG homozygous, and 1 was AG heterozygous. Notably, the proportion of germplasm carrying the long-grain GG genotype accounted for 22.7% of the total resources, with distinct distributions of 10% in indica, 69.6% in japonica, and 26.7% in “others”, suggesting significant differentiation at this locus between indica and japonica varieties (Table S2).

Comprehensive analysis of the grain length, width, and LWR data from 154 resources revealed that germplasm carrying the AA genotype at the GS3-Ex2 locus exhibited significantly longer grain length, shorter grain width, and higher LWR compared to those carrying the CC genotype (Figure 5D). In contrast, the two genotypes at the GW7-Pro locus did not exhibit significant differences in grain length, width, or LWR (Figure 5E). Given the significant distributional differences of this locus among subpopulations, we further examined its effects on grain shape across these subgroups. The results demonstrated that in japonica subpopulations, germplasms carrying the long-grain GG genotype exhibited significantly greater grain length compared to those with the AA genotype, while no significant differences were observed in grain width or LWR. In contrast, neither the indica subpopulations nor the “others” subgroup showed any significant variations in grain morphology traits (Table S5). The two alleles of GW7-Pro did not affect the primary effect of the GS3-Ex2 locus on grain length but exerted an additive effect on grain width and LWR. Specifically, when the GS3-Ex2 locus was the wider-grain CC genotype, germplasm carrying the GG genotype at the GW7-Pro locus was significantly longer in grain length and had a lower LWR compared to those with the AA genotype (Figure 5F).

### 2.6. GS3-Ex2 Has a Highly Significant Impact on Chalkiness-Related Traits, and the GW7-Pro Locus Exerts a Synergistic Effect

**Figure 6:**
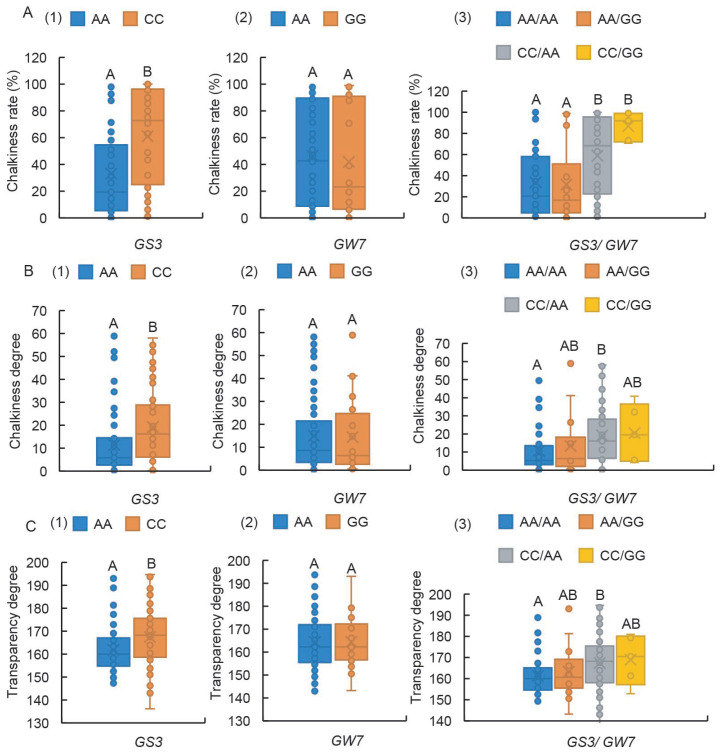
Boxplots showing phenotypic variation of haplotypes assayed by GS3-Ex2 and GW7-Pro in rice germplasm. (**A1**–**A3**) CR, (**B1**–**B3**) CD, (**C1**–**C3**) TD. Box edges represent the upper and lower quantiles, with median value shown as bold line in the box. Internal value points and outlier points are also displayed within the box. A–B indicate significant differences by Tukey’s *t*-tests (*p* < 0.01).

An investigation into the effects of two loci, *GS3* and *GW7*, on the appearance quality of rice, encompassing CD, CR, and TD (Table S4), revealed that germplasm carrying the GS3-Ex2-AA genotype showed markedly better performance than that with the GS3-Ex2-CC genotype in terms of CR, CD, and TD (Figure 6(A1,B1,C1)). Conversely, the two alleles of GW7-Pro did not significantly impact the visual quality of CR, CD, or TD (Figure 6(A2,B2,C2)). Notably, the combination of GS3-Ex2 and GW7-Pro loci did not alter the dominant role of GS3-Ex2 variation in influencing CR (Figure 6(A3)). However, only when GW7-Pro was in the AA genotype did the two alleles of GS3-Ex2 exhibit a highly significant effect on CD and TD (Figure 6(B3,C3)). These findings underscore the existence of a notable interaction between GS3-Ex2 and GW7-Pro loci in their influence on the appearance quality of rice grains.

### 2.7. GS3-Ex2 Locus Exerts an Effect in Controlling Both GC and ASV, While the GW7-Pro Locus Governs AC

**Figure 7:**
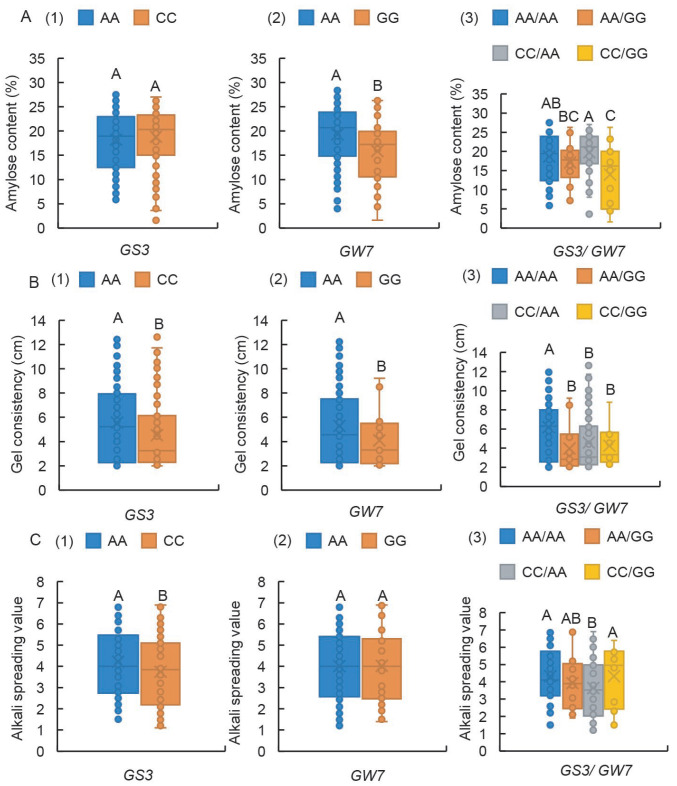
Boxplots showing phenotypic variation of haplotypes assayed by GS3-Ex2 and GW7-Pro in rice germplasm. (**A1**–**A3**) AC, (**B1**–**B3**) GC, (**C1**–**C3**) ASV. Box edges represent the upper and lower quantiles, with median value shown as bold line in the box. Internal value points and outlier points are also displayed within the box. A–C indicate significant differences by Tukey’s *t*-tests (*p* < 0.01).

Increasing the LWR can significantly enhance the appearance quality of rice grains, yet its impact on CEQ remains largely unexplored. Therefore, this study investigates the influence of the grain length-related genes *GS3* and *GW7* on CEQ (Table S4). For AC, the two genotypes at the GS3-Ex2 locus had no significant influence (Figure 7(A1)), whereas the germplasm carrying the GW7-Pro-AA genotype exhibited significantly higher AC values compared to those with the GW7-Pro-GG genotype (Figure 7(A2)). Notably, only when GS3-Ex2 was of the CC genotype were the extreme differences in AC between the two GW7-Pro alleles maintained (Figure 7(A3)).

Regarding GC, germplasm with the GS3-Ex2-AA and GW7-Pro-AA genotypes displayed significantly higher GC values than those with the GS3-Ex2-CC and GW7-Pro-GG genotypes, respectively (Figure 7(B1,B2)). Remarkably, germplasm concurrently harboring GS3-Ex2-AA and GW7-Pro-AA exhibited significantly higher GC than those with any of the other three genotypes (Figure 7(B3)).

In the case of ASV, the GS3-Ex2-AA genotype conferred significantly higher ASV than the GS3-Ex2-CC genotype (Figure 7(C1)). However, the two genotypes at the GW7-Pro locus did not significantly alter ASV (Figure 7(C2)). Specifically, only when GW7-Pro was of the AA genotype did the ASV difference between the two GS3-Ex2 alleles remain extreme (Figure 7(C3)). Collectively, these results indicated that as CEQ, the GS3-Ex2 locus exerts an effect in controlling both GC and ASV, while the GW7-Pro locus governs AC.

## 3. Discussion

Improving the appearance quality and CEQ of rice is central to rice quality breeding efforts, and several major genes involved in rice quality enhancement have been cloned and applied in breeding programs [26]. Previous studies utilizing transgenic technologies and constructing near-isogenic lines (NILs) have reported on the impacts of individual or several genes on rice quality under similar genetic backgrounds [27,28]. However, few studies have examined the roles of major genes in a genome-wide context across diverse rice germplasm resources. In this study, among 156 rice germplasm resources, we identified five loci, GS3-Ex2 and GW7-Pro associated with grain shape, and Wx-In1, Wx-Ex10, and SSIIa-Ex8 related to CEQ, as having extremely significant effects on rice quality.

### 3.1. Wx-In1, Wx-Ex10, and SSIIa-Ex8 Loci Exhibit Highly Significant Effects on Rice CEQ

The influence of different alleles of *Wx* and *SSIIa* on rice CEQ has been widely reported. In this study, we designed KASP markers targeting three critical sites in *Wx* and *SSIIa*, measured CEQ indicators in 156 germplasm resources encompassing both indica and japonica rice, and validated the usefulness of these markers (Figure 1 and Figure 2). The predominant control of AC by Wx-In1 is undeniable (Figure 3A). Furthermore, regardless of analyzing single-gene effects or two-gene and three-gene interactions, the influence of Wx-Ex10 on AC was consistently greater than that of SSIIa-Ex8 (Figure 3A). This can be attributed to two main reasons: first, the epistasis of Wx-In1 over SSIIa-Ex8 is not affected by Wx-Ex10, whereas the epistasis of Wx-In1 over Wx-Ex10 is modulated by SSIIa-Ex8 (Figure 3(A7)), indicating that Wx-In1 has an absolute major effect on SSIIa-Ex8 but its effect on Wx-Ex10 is influenced by other genes. Second, in the analysis of Wx-Ex10/SSIIa-Ex8 interactions, germplasm with the TT genotype at Wx-Ex10 consistently showed significantly higher AC than those with the CC genotype, regardless of SSIIa-Ex8 variations (Figure 3(A6)), suggesting a stronger influence of Wx-Ex10 on AC compared to SSIIa-Ex8. For AC, the effects of different differential loci follow the order of Wx-In1 > Wx-Ex10 > SSIIa-Ex8, which is entirely consistent with previous research findings that the different alleles of the *Wx* gene have a major regulatory role in controlling AC, while *SSIIa* also exerts an influence on AC [3].

While mainstream research often emphasizes the dominant role of CC versus TT variations at the Wx-Ex10 locus in governing GC [11], our study revealed that variations at Wx-In1 and SSIIa-Ex8 had more significant effects on GC, surpassing those of Wx-Ex10 (Figure 3B). This discrepancy could stem from the lack of independent analysis of indica, japonica, and “others” subspecies in our study, and the Wx-Ex10 locus displays significant evolutionary divergence (Table S2), potentially masking the primary effect of Wx-Ex10 on GC due to the combined effects of *Wx^a^* and *Wx^b^*. Previous studies reporting significant effects of Wx-Ex10 on GC were based on recombinant inbred lines (RILs) derived from indica parents carrying *Wx^a^* [11]. Additionally, when *Wx* exhibits the *Wx^a^* genotype, the CC and TT alleles at Wx-Ex10 directly determine low and high RVA values, another crucial indicator of CEQ, respectively [8]. The failure to independently analyze subspecies and the fact that *Wx* may not be the major gene controlling GC [3,4], may have obscured the effect of Wx-Ex10 on GC in our study. This highlights the need for further exploration and refinement of GC-controlling genes. Regarding the highly significant effect of SSIIa-Ex8 on GC, a possible explanation is that both *Wx* and *SSIIa* are located on chromosome 6, and their linkage and cosegregation allow the regulatory effect of Wx-In1 on GC to indirectly and significantly impact SSIIa-Ex8.

The GC/TT SNP effectively discriminated rice varieties with high or intermediate GT from those with low GT in approximately 90% of the cases studied [14,15,16]. This investigation also confirmed the primary effect of SSIIa-Ex8 on ASV (Figure 3C). Furthermore, it was observed that the impacts of Wx-In1 and Wx-Ex10 on ASV emerged only when SSIIa-Ex8 exhibited the TTTT genotype associated with high ASV (Figure 3C), suggesting intricate interactions among genes, whereby the function of Wx-In1 and Wx-Ex10 might be modulated by the SSIIa-Ex8 genotype in relation to ASV. The ultimate manifestation of rice quality is not solely determined by individual genes but rather shaped by a multitude of factors, including multiple genes and their interactions, as well as environmental conditions. Consequently, breeding practices necessitate a holistic approach that takes into account the combined effects of multiple genetic loci.

### 3.2. The Loci of Wx-In1, Wx-Ex10, and SSIIa-Ex8 Exhibit Highly Significant Effects on Chalkiness-Related Traits

Chalkiness often leads to potentially inferior eating quality in cooked rice grains [29]. Notably, chalkiness-related traits display a significant positive correlation with AC and a negative correlation with ASV and GC. Mutations or variations in the *Wx* gene are implicated in modifying AC, subsequently influencing grain transparency [8]. RNA interference (RNAi)-mediated repression of *SSIIa* slightly enhances chalkiness area and ratio, with minimal effects on white belly or white back [30]. In this study, we further investigated the effects of three loci from two genes on chalkiness-related traits and found that Wx-In1 > SSIIa-Ex8 > Wx-Ex10 in terms of their influence (Figure 4). Intriguingly, the significant effect of SSIIa-Ex8 on chalkiness-related traits was observed only when Wx-Ex10 was in its CC genotype, associated with superior appearance quality. Simultaneously, the significant effect of Wx-In1 on chalkiness-related traits was observed only when SSIIa-Ex8 was in its TTTT genotype, also associated with superior appearance quality. This finding, coupled with our previous conclusion that Wx-In1 and Wx-Ex10 impact ASV only when SSIIa-Ex8 is in its TTTT genotype associated with high ASV, underscores that the favorable genotypes of one or more genes can facilitate the efficacy of interacting genes when aiming to improve multiple traits simultaneously. In summary, these key loci involved in starch synthesis are equally crucial for rice grain appearance quality.

### 3.3. GS3-Ex2 and GW7-Pro Exert Highly Significant Effects on Rice Quality

*GS3* and *GW7* are two pivotal genes regulating grain length, with *GW7* and *GL7* being allelic variants of *Os07g0603300*. The copy number variation (CNV) at the GL7 locus, when combined with *GS3*, significantly enhances the appearance quality of rice grains [21]. Based on this, the present study investigated the impact of an 11-bp indel polymorphism in the promoter region of *GW7*, in conjunction with *GS3*, on rice grain appearance [20]. The role of GS3-Ex2 in determining grain shape is undeniable, and its different genotypes are evenly distributed across the indica and japonica subspecies. In contrast, the GW7-Pro locus exhibits limited influence on grain shape, with the long-grain GG genotype accounting for only 10% of the indica varieties tested but 69.6% of japonica varieties, indicating evolutionary divergence at this locus between subspecies (Figure 5). Given that indica varieties dominated our study population, this might have contributed to the insignificant effect of GW7-Pro on grain shape. When conducting subgroup analyses, the GW7-pro GG genotype significantly increased grain length in japonica, with a nonsignificant grain width and LWR increase. No effects were detected in indica or “other” subpopulations (Table S5). This evolutionary-diverged locus thus shows preferential utility for japonica grain shape breeding. Future research should include a sufficient number of both indica and japonica varieties to comprehensively evaluate the role of this locus in modulating grain appearance quality within each subspecies. Despite the nonsignificant effect of the GW7-Pro locus on grain shape variation, its additive effect with *GS3* on several quality indicators suggests that this locus, similar to the CNV at GL7, can influence rice appearance quality (Figure 5).

There exists a significant correlation between grain shape and chalkiness-related traits. Specifically, chalkiness percentage is negatively correlated with grain length and length-to-width ratio, while positively correlated with grain width and weight [31,32]. Grain shape is a complex trait. Given that GW7 can simultaneously regulate grain length and appearance quality, this study focuses on the collaborative regulation of chalkiness-related traits by GW7 and GS3. Our study demonstrates that the GS3-Ex2 locus exerts highly significant effects on CD, TD, and CR. In contrast, the GW7-Pro locus, whose influence on CD, TD, and CR aligns with its effect on grain shape, exhibits insignificant individual effects but can interact with the GS3-Ex2 locus to produce an effect (Figure 6). A possible reason is that multiple genes regulating grain length, width, and thickness have been identified, among which the grain width gene *GW8* directly regulates *GW7* [33]. This complex interplay among genes results in the interaction of multiple gene genotypes masking the effect of *GW7* when analyzed in natural populations.

Furthermore, we investigated the impact of both GS3-Ex2 and GW7-Pro loci on CEQ. GS3-Ex2 had no significant effect on AC but significantly influenced GC and ASV. The AA genotype associated with long grains exhibited high GC and ASV (Figure 7), confirming that increased grain length can enhance CEQ, thereby improving rice quality. Conversely, GW7-Pro had no significant effect on ASV but significantly impacted AC and GC. The GG genotype associated with long grains reduced AC, potentially enhancing rice palatability, yet simultaneously decreased GC (Figure 7). This may be attributed to the unequal representation of this locus in indica and japonica subspecies, leading to distorted effects on rice quality when studying a combined indica-japonica population. This underscores the need for expanded population sizes and subgroup analyses for loci with pronounced evolutionary divergence between subspecies.

In summary, our findings reveal a close relationship among grain shape, chalkiness-related traits, and CEQ in rice. The GS3-Ex2 and GW7-Pro loci associated with grain shape, along with Wx-In1, Wx-Ex10, and SSIIa-Ex8 loci related to CEQ, collectively influence rice quality, providing a research foundation and molecular markers for rice quality improvement. While the current study focused on characterizing haplotype effects in rice resources, we acknowledge that formal statistical modeling of gene-gene interactions (through additive models or interaction terms) would provide deeper insights into epistatic mechanisms. Furthermore, although this study obtained conclusions that largely aligned with expectations using the available data, it should be noted that rice quality traits are quantitative traits, and both the appearance quality indicators and CEQ metrics are highly susceptible to environmental influences. The reliability of the results remains constrained by the fact that the data were collected from only a single location and growing season. Future studies should expand the population size and conduct two-year replicated trials in both Hainan and Hangzhou, China, to acquire more comprehensive data for in-depth analysis. This approach would effectively mitigate the impact of environmental factors on the results, enhance the detection power of interaction effects, and particularly facilitate the identification of rare allele combinations whose effects were underpowered in the current dataset due to limited statistical power.

## 4. Materials and Methods

### 4.1. Plant Materials

The 156 rice resources were planted simultaneously in an experimental field at the Zhejiang Academy of Agricultural Sciences in Hangzhou, China. The mature grains were dehulled, and the brown and milled kernels were used as plant materials for this study.

### 4.2. DNA Extraction and Kompetitive Allele-Specific PCR (KASP) Marker Assay

Genomic DNA from fresh young leaves of rice was extracted using the CTAB method. All primers were synthesized at Tsingke Biotech (Beijing, China). For KASP primer design, the functional SNPs for *Wx*, *SSIIa*, *GS3*, and *GW7* were targeted, based on http://www.snpway.com:8339/, accessed on 3 April 2024. The primer sequences are provided in Table S1. The PCR protocol for KASP was as follows: 5 µL (20–30 ng/µL) DNA, 0.14 µL primer mixture, and 5 µL 2× KASP Master mixture (KBS-1016-002, LGC, Hoddeston, UK). The cycling regime was as follows: 94 °C for 15 min, 10 touchdown cycles (94 °C for 20 s, touchdown at 61 °C initially, and decreasing by 0.6 °C per cycle for 60 s), followed by 28 additional cycles of annealing (95 °C for 20 s, 55 °C for 60 s). The PCR products were detected using the RT-PCR system (CFX-96, BioRad ^®^, Hercules, CA, USA).

### 4.3. CEQ Measurement

The AC was assessed following the method described by [3]. The ASV was determined using the alkali digestion test. A duplicate set of six whole-milled kernels without cracks was selected and placed in a plastic box (5 × 5 × 2.5 cm). Then, 10 mL of 1.7% KOH solution was added. The samples were arranged to provide enough space between kernels to allow for spreading. The boxes were covered and incubated for 23 h in a 30 °C oven. The starchy endosperm was visually rated based on a seven-point numerical spreading scale, which serves as a standard evaluation system for rice. Based on the ASV score, rice grains can be classified into four groups: high (1–2), high-intermediate (3), intermediate (4–5), and low (6–7). GC was measured using flour (100 mg) from all samples, weighed in duplicate into 13 mm × 100 mm tubes, and 200 µL of ethyl alcohol (95%), containing 0.025% thymol blue, was added to each tube along with 2 mL of 1 M KOH. The tubes were then placed in a vigorously boiling water bath for 8 min. After the tubes were removed from the water bath, they were held at room temperature for 5 min and then cooled in an ice water bath for 20 min. Following this, the tubes were laid horizontally on a lightbox on top of graphing paper, and after 1 h, the distance that the gel migrated in the tube was measured.

### 4.4. Grain Shape Measurement

The grain length, grain width, and length-to-width ratio were measured utilizing a seed-testing instrument.

### 4.5. Measurement of Chalkiness-Related Traits

Chalkiness rate (CR): Randomly select 100 whole polished grains from the sample and place them on a glass plate. Under a condenser lamp, visually inspect the grains to segregate those with chalkiness. Calculate the percentage of chalky grains using the following formula: CR (%) = (Number of Chalky Grains/Total Number of Grains) × 100. This process is repeated once, and the average of the two measurements is taken as the final result.

Chalkiness degree (CD) and translucent degree (TD): Randomly select a subset of whole polished grains from the sample and place them on a dedicated scanner’s attached black placement board. Subsequently, the grains are analyzed using the software, chalkinessV2.0, for quantification of CD and TD.

## 5. Conclusions

In summary, we designed five KASP markers based on important allelic sites located on four major genes (*Wx*, *SSIIa*, *GS3*, and *GW7*) that regulate CEQ and appearance quality of rice. These markers were used to genotype 156 germplasm resources. We then investigated the effects of individual and combined allelic genotypes on the rice quality of these germplasm resources. The results showed that the Wx-In1 locus exerts a major effect in controlling both amylose content and gel consistency, while the SSIIa-Ex8 locus primarily governs alkali spreading value. The impact on chalkiness-related traits follows the hierarchy of Wx-In1 > SSIIa-Ex8 > Wx-Ex10. GS3-Ex2 has a highly significant impact on chalkiness-related traits, and the GW7-Pro locus exerts a synergistic effect. The GS3-Ex2 locus exerts an effect in controlling both gel consistency and alkali spreading value, while the GW7-Pro locus governs amylose content. These markers can be used in the future for molecular marker-assisted breeding.

## Data Availability

The original contributions presented in this study are included in the article and Supplementary Materials. Further inquiries can be directed to the corresponding author.

## Supplementary Materials

The following supporting information can be downloaded at https://www.mdpi.com/article/10.3390/ijms26146726/s1.
